# Acoustic Limescale Layer and Temperature Measurement in Ultrasonic Flow Meters

**DOI:** 10.3390/s22176648

**Published:** 2022-09-02

**Authors:** Johannes Landskron, Florian Dötzer, Andreas Benkert, Michael Mayle, Klaus Stefan Drese

**Affiliations:** 1 ISAT—Institute of Sensor and Actuator Technology, Coburg University of Applied Sciences and Arts, 96450 Coburg, Germany; 2 Diehl Metering, 91522 Ansbach, Germany

**Keywords:** flow metering, ultrasound, guided acoustic waves, Lamb waves, limescale layers, temperature compensation, FEM simulation, predictive maintenance, product lifetime extension

## Abstract

Guided acoustic waves are commonly used in domestic water meters to measure the flow rate. The accuracy of this measurement method is affected by factors such as variations in temperature and limescale deposition inside of the pipe. In this work, a new approach using signals from different sound propagation paths is used to determine these quantities and allow for subsequent compensation. This method evaluates the different propagation times of guided Lamb waves in flow measurement applications. A finite element method-based model is used to identify the calibration curves for the device under test. The simulated dependencies on temperature and layer thickness are validated by experimental data. Finally, a test on simulated data with varying temperatures and limescale depositions proves that this method can be used to separate both effects. Based on these values, a flow measurement correction scheme can be derived that provides an improved resolution of guided acoustic wave-based flow meters.

## 1. Introduction

Hard water forms limescale layers in industrial and domestic piping [1,2,3]. Blocked pipes or the mechanical failure of valves, boilers, faucets, and flow meters are caused by the progressive layer growth followed by high costs for maintenance [4]. The avoidance of limescale layers by ion exchangers or chemical additives incurs additional costs as well [5]. Therefore, this method is hardly used and the formation of layers is accepted in most applications. To overcome the risks of total failure, various methods for detection, characterization, inhibition, and removal of limescale layers are investigated in many studies [3,6,7,8,9,10,11,12]. Potential approaches are based on optical, chemical, thermal, or acoustic methods.

In recent years, flow meters without moving components, known as solid-state water meters or static meters, have been established [13,14]. However, the accuracy of these ultrasonic-based water meters decreases with time due to the formation of limescale layers. To correct the deviations, online monitoring of the depositions in the sensors must be integrated. Unfortunately, methods such as infrared thermography [11] are not suitable for low-cost permanent online monitoring in home applications such as domestic water meters. In particular, the determination of the layer thickness using the flow meter-integrated measurement equipment would be of great advantage. Water meters using guided acoustic waves (GAWs) are one type of ultrasonic sensor which may benefit from this feature. Besides flow meters, GAWs are commonly used in nondestructive testing and layer monitoring applications [15,16,17,18,19,20,21,22,23,24,25]. The detection of different kinds of layers, e.g., limescale layers or biofilms using GAWs, has previously been investigated by the authors [26,27,28].

Furthermore, temperature fluctuations complicate the acoustic flow measurement resulting in additional time shifts in the measured data [24,29,30,31,32]. Temperature effects are often large compared to the effects of thin limescale layers. Therefore, temperature compensation techniques are mandatory in such applications [33]. Whereas the deployment of a separate temperature probe is the most straightforward approach, it requires additional hardware and only probes the temperature at a single point, normally on the outer surface. This local point measurement does not necessarily provide sufficient information for successful temperature compensation of GAWs since there can be temperature gradients within the flow meter.

The goal of this work is the detection of limescale layers with integrated temperature compensation in GAW-based flow meters. Only already available hardware in the sensor is used. The method is demonstrated by a specific transducer arrangement evaluating sound signals of two different propagation paths. Based on finite element method (FEM) simulation data supported by experiments, it was possible to successfully determine the addressed quantities.

## 2. Materials and Methods

### 2.1. Fundamentals of the Lamb Wave-Based Flow Meter

**Figure 1:**
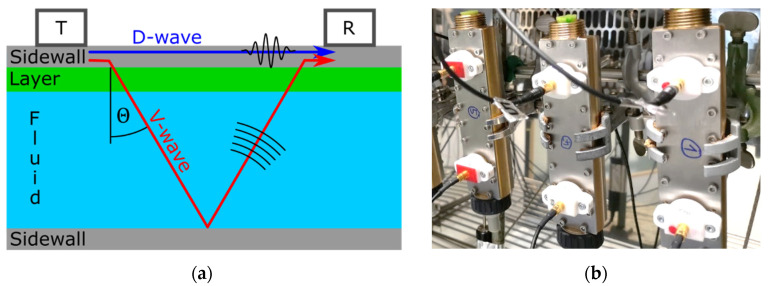
(**a**) Schematics of the setup including an ultrasound transmitter (T), receiver (R), the direct (D) and indirect (V) propagation paths in the Lamb wave flow meter as investigated in this article. (**b**) The studied ultrasonic flow meters in the climate chamber.

In the present work, a prototypical flow meter sensor with a rectangular cross-section (*W* × *H* = 10 mm × 15 mm) was investigated. Two ultrasound transducers were attached outside on one of the sidewalls, which we denote the “upper” sidewall in the following, acting as a transmitter (T) and receiver (R) as shown in Figure 1a. The sidewalls were made of stainless steel 1.4571 [34] with a thickness of 1.5 mm and a distance between the transducers of 90 mm. Limescale layers were applied on the inside of this very same sidewall. The transducers were designed in such a way that they would predominantly excite and detect a specific type of GAW. The chosen GAW was the antisymmetric A0-mode Lamb wave [35,36,37]. A typical attribute of Lamb waves is their dispersive behavior. The excitation of the dominant A0-mode was verified by a scanning laser Doppler vibrometer (Polytec GmbH, PSV-400-M, Waldbronn, Germany).

The measurement principle was as follows. The Lamb wave, which was excited by the transmitter T, propagated along the upper sidewall. While doing so, it radiated a pressure wave into the fluid, which made it a so-called leaky Lamb wave. It is this leaked pressure wave that is commonly used to measure flow velocities in the fluid by evaluating the difference in propagation time in forward and backward directions [38]. The pressure wave was radiated at the so-called Rayleigh angle Θ, which is defined by the phase velocities $v_{L}$ of the Lamb wave and $v_{p}\;$ of the pressure wave in the fluid, respectively, as $\Theta =\arcsin\left(v_{p}/v_{L}\right)$. The pressure wave was reflected at the lower sidewall of the flow channel. Upon return at the upper sidewall, this reflected pressure wave in turn excited a Lamb wave which consequently contributed to the signal at the receiver. In the chosen configuration, not all of the Lamb wave’s initial energy had been radiated into the fluid when it reached the receiver. In this case, contributions from two propagation paths were observed in the received signal: the initial but attenuated wave traveling in a straight, direct path along the sidewall to the receiver (direct, D-wave) and the above-described radiated parts traveling through the fluid (along a V-shaped path, V-wave). The corresponding propagation times we denote as $t_{D}$ and $t_{V}$, respectively.

Next, we considered two influencing factors on the flow measurement with the above outlined guided acoustic wave principle: limescale layers and temperature. A limescale layer caused a slight change in phase velocity of the Lamb wave in the sidewall [26]. This also modified the Rayleigh angle and therefore the path through the fluid. Additionally, for a given volume flow Q, the layers on the sidewalls led to a higher flow velocity v by means of a decreased cross-sectional area A: v=Q/A. If not accounted for, both mechanisms reduced the accuracy of the flow measurement by their respective influence on the propagation times of the V-wave. At the same time, these changes in propagation times were exploited to estimate the thickness of the limescale layer, which was one of the goals of the present work.

The other considered influence factor on the propagation times $t_{D}$ and $t_{V}$ was temperature variation. For a start, the geometric parameters such as the distance between the two parallel sidewalls and the one between the transducers were modified by thermal expansion. Additionally, the thickness of the sidewalls was changed, which in turn altered the phase velocity of the Lamb wave. The latter also was influenced by the temperature dependence of the material’s density and Young’s modulus. Similarly, the sound velocity and density of water varied with temperature, leading to modified propagation and radiation characteristics. Finally, the limescale layer itself may exhibit temperature-dependent material properties, as well.

### 2.2. Experimental Methods

The strategy to measure the layer thickness and temperature presented in this paper is based on simulation data and is outlined in a later section. Nevertheless, experimental data are necessary to adapt the material’s mechanical parameters used for the simulations and to validate the results. In this section, we provide an overview of our experimental methods to generate this data.

The first and most important step was to produce the limescale layers. Two strategies were pursued. In the first one, the limescale layers were produced by an application-orientated flow loop with hardened water to which the sensors were exposed for up to 6 months. Three such “accelerated aged” sensors were prepared for the current investigation. Thicknesses in the range of 10 µm to 100 µm were achieved. In a second approach, limescale layers were produced by means of evaporating a saturated solution of water. This yielded limescale layers with thicknesses of about 150 μm. These layers were subsequently removed step-by-step. Preceding experiments had shown that the application of a mixture of 94% water and 6% acetic acid for 30 s reproducibly reduced 10 μm from the surface of these limescale layers. This allowed performing measurements at several intermediate layer thicknesses starting from a single applied limescale layer. In each step, the layer thicknesses were characterized by disassembling the sensor and a subsequent inspection with a confocal laser scanning microscope (Keyence Deutschland GmbH, VK-X200, Neu-Isenburg, Germany).

**Figure 2:**
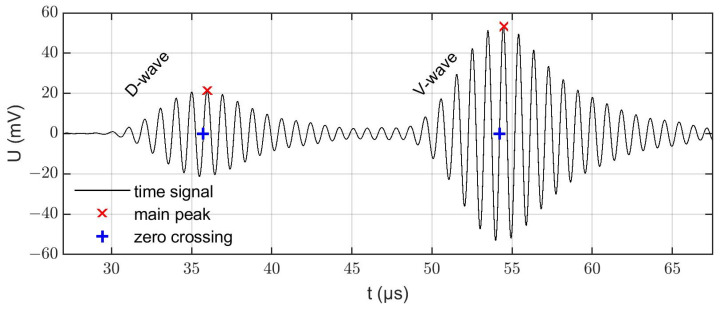
Measured voltage signal (U), marked main peak and zero crossing of the D-wave (29–45 µs) and the V-wave (48–65 µs) contributions, respectively.

For the acoustic measurements, the sensors were operated with an input signal for the transmitter which is given by a Hann function windowed 1 MHz sine burst with a duration of ten periods and a signal amplitude of 3 V_pp_. It was provided by an arbitrary waveform generator (Agilent Technologies Deutschland GmbH, 33521A, Waldbronn, Germany). At the receiver’s side, the signal was recorded by an oscilloscope (Teledyne LeCroy GmbH, WaveRunner 604 Zi, Heidelberg, Germany). A typical received signal is displayed in Figure 2. The contributions from the D and V propagation paths are clearly distinguishable and the respective signal amplitudes are of the same order of magnitude.

There are various ways to deduce an ultrasonic propagation time from such signals [39]. As we were focusing in the present work on methodical aspects rather than on the highest achievable accuracy, we chose one of the conceptually simplest methods for propagation time determination: the zero-crossing algorithm. That is, the propagation times $t_{D}$ and $t_{V}\;$ were determined by evaluating the time of the zero crossings (Figure 2, blue pluses) right before the main peak (Figure 2, red crosses) of the two waves, respectively. The samples around the zero crossing were interpolated linearly to achieve a higher resolution than the experimental sampling rate provided. One caveat of this method was that temperature and limescale layers could also influence the envelope and amplitude of the measurement signal. It could happen that a decreasing amplitude of the initial main peak resulted in a new maximum peak at a different position in the time signal, which in turn would have led to a wrong zero crossing to be analyzed. To avoid measurement errors caused by this effect, tracking the initially selected peak was essential.

The effect of temperature on $t_{D}$ and $t_{V}$ was investigated by placing the flow meter sensor setup in a climatic chamber (CTS Clima Temperatur Systeme GmbH, C-60/200 Ex, Hechingen, Germany) which provided well-defined temperatures ranging from −60 °C to 150 °C. Measurements were performed at regular intervals during the temperature cycle, ranging from 5 °C to 50 °C at ambient humidity. Using a temperature probe placed inside the sensor, the actual temperature of the water inside the flow channel was tracked.

### 2.3. Simulation Methods

Although experimental data are important for validation aspects, noise-free simulation data with precisely known input parameters proved more useful to derive a new method for limescale layer detection and characterization. To generate such acoustic signals without noise and other imperfections, FEM simulations were performed.

The simulated signals were generated using COMSOL Multiphysics (version 5.5) in a two-dimensional transient study. It contained the upper sidewall, transducers, limescale layer, water domain, and a sound-hard boundary (representing the lower sidewall) as shown in Figure 1. Suitable boundary conditions suppressed interfering reflections from the borders of the computational domain. Furthermore, piezoelectric transducers were modeled as mode selective transmitters and receivers on the upper sidewall. The physical and acoustic properties of water were well known and were predefined in the COMSOL library. For the steel sidewall, the material properties from the datasheet were used [34]. For the piezoelectric transducer, experimentally fitted material parameters were applied [40].

**Figure 3:**
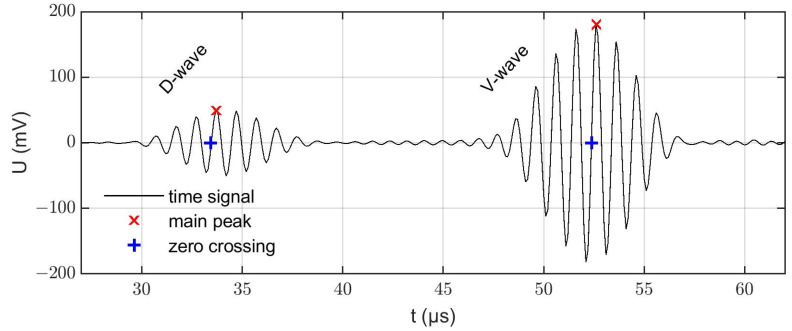
Simulated voltage signal (U) at the receiver at 20 °C and without a limescale layer with D-wave (29–39 µs) and V-wave (47–57 µs) contributions.

As in the experiments, the excitation was performed on the transmitter by a 10-period sine wave signal at 1 MHz windowed by a Hann function. In the simulations, a normalized amplitude of 1 V_pp_ was used. For the postprocessing, the voltage curves on the receiver were stored and evaluated. Figure 3 shows a simulated time signal including the D-wave and V-wave. The same zero-crossing algorithm as for the experimental data was used to track the propagation times of the D-wave ($t_{D}$) and V-wave ($t_{V}$).

In the simulations, the thickness of the limescale layer and the temperature of the system were varied to identify their effects on $t_{D}$ and $t_{V}$. The change in layer thickness could be implemented by changing the height of the limescale layer domain. To model the temperature effects, the temperature-induced expansion of the system and changes in the material properties had to be considered. To this end, the temperature-dependent quantities from the steel datasheet were linearly interpolated. The temperature behavior of water in a range from 0 °C to 100 °C was well known and part of the COMSOL material library. The material properties for the limescale layers, however, were not known a priori and had to be determined iteratively in the first simulations. Starting from literature values, the parameters of the limescale layer were therefore optimized until the layer sensitivity of the D-wave propagation times in the simulations matched the one from the experiments. The result from a laser acoustic measurement of the mechanical parameters of the limescale layer confirmed the parameters obtained by the adaption of the simulation. Due to the lack of significant data on the temperature dependence of limescale layers and the difficulty of its experimental investigation, these effects were currently not implemented in the simulation of this paper.

## 3. Results

In this section, we present and explain the data from our experiments and simulations. Based on these data, an evaluation algorithm was derived that allowed the determination of the limescale layer thickness and the temperature of the medium.

### 3.1. Experimental Results

**Figure 4:**
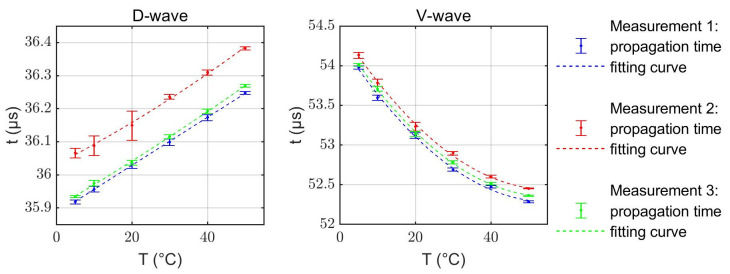
Experimentally measured propagation times $t_{D}$ and $t_{V}$ depending on the temperature in a range from 5 °C to 50 °C. The vertical offset in the curves is related to variations in the transducer attachment on the different sensors or variations in limescale layer thickness.

The data from the three “accelerated aged” sensors were studied first. The obtained propagation times at different stabilized temperatures for the three sensors are shown in Figure 4. The experimental data are closely approximated by the shown quadratic fit functions. Although minor deviations of the absolute propagation times are present, the temperature dependencies are very similar for all tested sensors with different layer thicknesses. Hence, the temperature dependence of the propagation times only negligibly depend on the layer thickness. Changes in propagation times along the V-path are predominantly determined by the changes in the sound velocity in water and exhibit a strong, nonlinear temperature dependence (ranging from about −100 ns/K to 0 ns/K), whereas the D-path is less sensitive and shows a close to linear behavior (about +8 ns/K).

**Figure 5:**
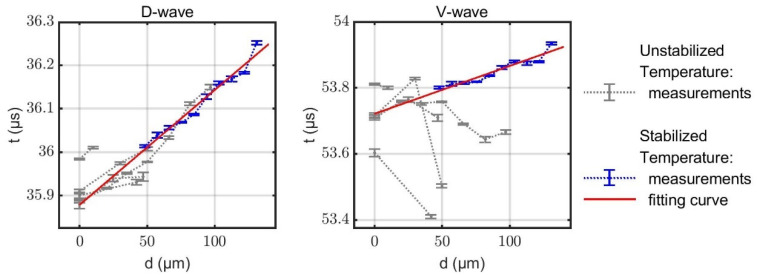
Experimentally measured propagation times $t_{D}$ and $t_{V}$ as a function of the limescale layer thickness. A total of six layers are incrementally removed with acid and measurements are made at intermediate steps. Attempts are made to compensate for temperature fluctuations according to the previous results. In the last measurement (red), the temperature was kept as constant as possible, leading to an improved result.

The second set of data stems from a measurement series with step-by-step decreasing thickness of the limescale layers. As the temperature inside the sensor was monitored, the data can be corrected for temperature effects using the preceding results of the investigations on the temperature dependencies. The resulting temperature compensated propagation times along the D- and V-paths are displayed in Figure 5. The first results (grey data points) show that stable temperature control is crucial for meaningful data, especially for the highly temperature-sensitive V-wave (cf. Figure 4). Hence an improved temperature stabilization was introduced for one measurement series, which is shown in blue. The corresponding fitting curves are printed in red.

The propagation time $t_{D}$ shows a clear trend, which is similar for each measurement series. The slope can be estimated to be 2.5–3.0 ns/μm. In contrast, no generally valid trend can be observed for the unstabilized data of the V-path. Rather, large variations among the different measurement series are evident. This we attribute to the strong temperature sensitivity of the V-wave. Moreover, inhomogeneities of the spatial temperature distribution may lead to refraction, alteration of the propagation path, and consequently to variations in the propagation times. Measuring the water temperature at only one position therefore may not be sufficient to correct all temperature-induced deviations and could explain the erratic behavior of $t_{V}$ despite the temperature compensation. As a result, during the measurement of the last test specimen, the temperature was kept constant to within 1 °C (compared to variations of up to 5 °C during the previous measurement series), yielding a less erratic behavior of $t_{V}$ (blue line).

### 3.2. Simulation Results

**Figure 6:**
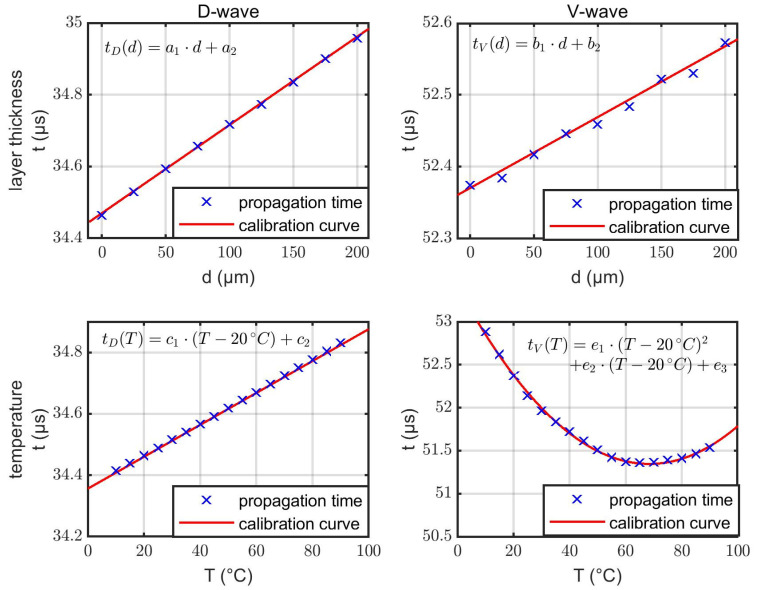
Evaluated propagation times $t_{D}$ and $t_{V}$ (blue crosses) related to changes in layer thickness d and temperature T without mutual interdependencies. In addition to the simulation results, fitted calibration curves (red lines) including their mathematical representations are shown.

Next, we present the simulation results which provide the data basis for the derivation of the evaluation algorithm in the next subsection. Figure 6 shows the simulated dependencies of $t_{D}$ and $t_{V}$ according to limescale layers and temperature effects. No mutual interdependencies are assumed, i.e., the temperature is kept constant while varying the layer thickness and vice versa. Accordingly, one study varies the layer thickness from 0 µm up to 200 µm in 25 µm steps at 20 °C. The second study increases the temperature from 10 °C up to 90 °C in steps of 5 °C without any limescale layers at all.

As outlined in the Methods section, the material parameters of limescale are obtained by an optimization procedure to the experimental results from the previous subsection. There is no other free parameter in the current simulation to adapt the simulation results further. After the fitting of the limescale material data, an increasing limescale layer thickness in the simulation results in linearly increasing propagation times $t_{D}$ and $t_{V}$, just as observed in the experiments. Temperature effects exhibit similar behavior as in the experiments as well: a linear dependence for the D-wave and quadratic dependence for the V-wave.

**Figure 7:**
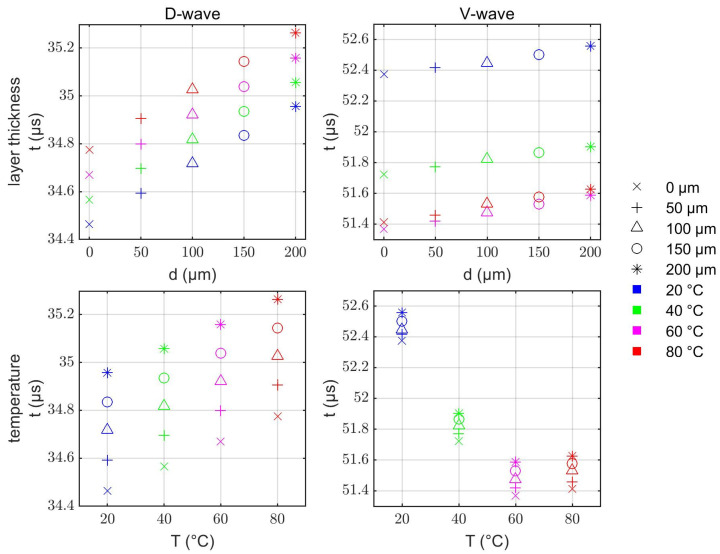
Evaluated propagation times $t_{D}$ and $t_{V}$ related to changes in layer thickness (d) and temperature (T). The trends show independent sensitivities to the individual parameters. These data points are used for testing the evaluation algorithm in Section 3.3.

In the next step, the interaction between limescale layers and temperature variation was investigated. Simulations at various combinations of different temperatures and layer thicknesses showed that there is only a negligible influence of the layer thickness on the temperature behavior and vice versa (Figure 7). This agrees with the previous experimental results shown in Figure 4.

Based on these results, the two effects are approximated to contribute separately to the total propagation times $t_{D}\left(d,T\right)$ and $t_{V}\left(d,T\right)$ according to

(1) $$t_{D/V}\left(d,T\right)=t_{D/V}\left(d\right)+t_{D/V}\left(T\right)-\frac{1}{2}\left(t_{D/V}\left(0\;\mu m\right)+t_{D/V}\left(20\;{}^{\circ}C\right)\right)$$

where

(2) $$t_{D}\left(d\right)=a_{1}d+a_{2}\;t_{V}\left(d\right)=b_{1}d+b_{2}\;t_{D}\left(T\right)=c_{1}\left(T-20\;{}^{\circ}C\right)+c_{2}\;t_{V}\left(T\right)=e_{1}{\left(T-20\;{}^{\circ}C\right)}^{2}+e_{2}\left(T-20\;{}^{\circ}C\right)+e_{3}$$

are the fitted representations of simulated behavior of the propagation times $t_{D/V}$ for D- and V-waves as a function of layer thickness d and temperature T, respectively. The last term in Equation (1) compensates the double counting of the constant contribution to the propagation time.

### 3.3. Evaluation Algorithm

Based on the previous simulation results, an evaluation algorithm for the layer thickness on the sidewall and the temperature of the system was developed. The evaluation was based on the simulated propagation times $t_{D}$ and $t_{V}$. The data from Figure 6 was used to calibrate the system. The parameters of the specified calibration curves (cf. Equation (2)) are listed in Table 1. Normally, the calibration curves should give the same result for identical input parameters. We expected $t_{D/V}\left(0\;µm\right)=t_{D/V}\left(20\;{}^{\circ}C\right)$ because T=20 °C and d=0 μm were assumed for $t_{D/V}\left(d\right)$ and $t_{D/V}\left(T\right)$, respectively. Equivalently, this means $a_{2}=c_{2}$ and $b_{2}=e_{3}$. As we did not restrict our fitting procedure to this boundary condition, one found slight deviations from that as shown in Table 1. This small offset was fixed by the last term in Equation (1) which effectively employed an average of the constant contributions.

By combining the two equations for the D-wave from Equation (2) with the Formula (1), Equation (3) can be derived. This equation shows the combined dependence on layer thickness *d* and temperature *T*. It behaves linearly in *d* and *T*. The equations for the V-wave could be used in the very same way, resulting in Equation (4). In contrast to Equation (3), the temperature effect is now quadratic.

(3) $$t_{D}\left(d,T\right)=a_{1}\cdot d+c_{1}\cdot \left(T-20\;{}^{\circ}C\right)+k_{D},$$

(4) $$t_{V}\left(d,T\right)=b_{1}\cdot d+e_{1}\cdot {\left(T-20\;{}^{\circ}C\right)}^{2}+e_{2}\cdot \left(T-20\;{}^{\circ}C\right)+k_{V}.$$

In the above equations we abbreviated the constant contributions for simplicity:

(5) $$k_{D}=\frac{a_{2}+c_{2}}{2},$$

(6) $$k_{V}=\frac{b_{2}+e_{3}}{2}.$$

Next, Equation (3) can be solved for the layer thickness *d*. This gives a function of the temperature *T* and the propagation time $t_{D}\left(d,T\right)$ of the D-wave:

(7) $$d=-\frac{c_{1}}{a_{1}}\cdot \left(T-20\;{}^{\circ}C\right)-\frac{k_{d}}{a_{1}}+\frac{t_{D}\left(d,T\right)}{a_{1}}$$

We can now take this expression to substitute *d* in Equation (4) and solve for the temperature T. We obtain Equation (8) which is quadratic in *T:*

(8) $$\begin{array}{cc} 0= & \left[e_{1}\right]\cdot T^{2}+\\ & \left[e_{2}-e_{1}\cdot 2\cdot \left(20\;{}^{\circ}C\right)-\frac{b_{1}\cdot c_{1}}{a_{1}}\right]\cdot T+\\ & \left[k_{V}-t_{V}\left(d,T\right)+e_{1}\cdot {\left(20\;{}^{\circ}C\right)}^{2}-e_{2}\cdot \left(20\;{}^{\circ}C\right)+\frac{b_{1}\cdot t_{D}\left(d,T\right)}{a_{1}}-\frac{b_{1}\cdot k_{D}}{a_{1}}+\frac{b_{1}\cdot c_{1}}{a_{1}}\cdot \left(20\;{}^{\circ}C\right)\right] \end{array}$$

Both Equations (7) and (8) still contain the propagation times $t_{D/V}\left(d,T\right)$ which, of course, depend on d and T themselves. For a given measurement, however, they are mere constants that are derived from the measured signal as outlined in the Method section: $t_{D/V}\left(d,T\right)\to \;t_{D/V}$. Hence, from Equation (7) the layer thickness can be directly computed with the parameters given in Table 1 and the measured propagation time $t_{D}$ if the temperature T is known. In this case, only the D-wave contributes to this determination of the layer thickness.

If the temperature T is not known a priori, the evaluation scheme becomes more complicated. In principle, the temperature can be deduced from Equation (8). However, since Equation (8) is quadratic in *T*, there are two possible solutions $T_{1/2}$ that both satisfy the equation. By inserting $t_{D}$, $t_{V}$, and $T_{1}$ or $T_{2}$, respectively, into Equation (7) we obtain the two pairs of values $\left(T_{1}|d_{1}\right)$ and $\left(T_{2}|d_{2}\right)$ and we need to choose the physical one. Ultrasonic measurements usually are performed on very short timescales and with high repetition rates, whereas temperatures only change slowly and limescale layers grow even slower. Therefore, it is possible to select the correct solution from the two available pairs of values by tracking the values determined in the preceding measurements. For the very first measurements of sensor it is trivial—there is no limescale layer, hence the solution with d=0 μm can be selected.

**Figure 8:**
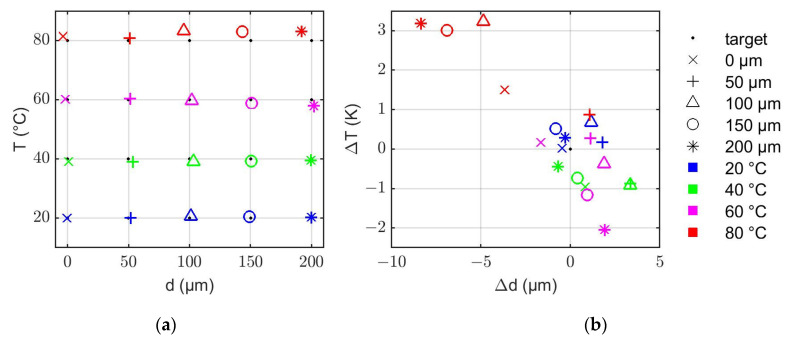
(**a**) Test grid with the evaluated values for the layer thickness *d* and temperature *T* based on the simulated propagation times $t_{D}$ and $t_{V}$ displayed in Figure 7. (**b**) Absolute deviation of the evaluated data from the test grid.

The above-described algorithm was validated by means of the simulation results shown in Figure 7. To this end, the results of the algorithm were compared to the actual layer thicknesses and temperatures that were used for the simulations. Figure 8a shows that the algorithm developed in this work can successfully evaluate the layer thicknesses and temperatures of the system. All solution pairs determined from the propagation times deviate only slightly from the test grid. Figure 8b shows the corresponding absolute errors. Here it can be observed that the deviations are <±4 µm and <±1.5 K in most cases. Only for the combinations of high temperatures (60 °C and 80 °C) and high layer thicknesses (100 µm–200 µm) larger deviations occurred. This is most likely related to the small temperature sensitivity of the V-wave around the minimum of the curve between 60 °C and 80 °C (cf. Figure 6). In this study, the maximum errors are −8.4 µm (@ 80 °C and 200 µm) and +3.2 K (@ 80 °C and 100 µm).

## 4. Discussion

The new evaluation method is based and tested exclusively on simulation data. Consequently, we had to ensure that the simulation represents the investigated mechanism as well as possible. The required parameters for the steel tube and the water domain were well known. The material parameters for limescale were iteratively adjusted to match the experimental results. Finally, the simulation results have shown the same trendlines for an increasing layer thickness as observed in the experiments. For the acoustic parameters of limescale, the sensitivity to changes in temperature is still unknown. An evaluation of the experimental data did not provide any significant insight. Nevertheless, the simulations reproduce the experimental trendlines, suggesting that the observable time shifts were caused by steel and water predominantly. Furthermore, the convergence of all simulations was always controlled. All calculation errors of the simulations are small compared to the obtained precision of the evaluation algorithm and can therefore be neglected.

In the laboratory, it was possible to produce layers much faster than is typical for domestic pipes. The characterization of these layers indicated varying microstructures and mechanical properties. In this study, an invasive optical method was used for layer thickness analysis. This procedure involves the risk that parts of the limescale layer may flake off during disassembly. In this case, a characterization of the layer’s thickness was no longer possible and the measurement series had to be aborted.

To increase the accuracy of the algorithm’s calibration curves, further interpolation points could be calculated or measured experimentally in future studies. This might reduce the evaluation error caused by the averaged $k_{D}$ and $k_{V}$. If necessary, slight modifications to the calibration curves postulated in Figure 6 will be conceivable with a larger and more realistic data set. For example, small oscillating components could improve the accuracy of the calibration curve for the layer sensitivity of the V-wave. However, this tends to further complicate the evaluation procedure and the correct solution might have to be selected from a larger set of possible solutions. Although this is not a fundamental problem, it might prove impractical in domestic water meters, where the available computational power will be limited.

## 5. Outlook and Conclusions

The next step is to validate the algorithm by experimentally generated test cases. The most critical aspect will be the production and characterization of realistic limescale layers. Since there is a risk of flaking during the disassembly process, the actually used microscopic methods are not suitable for the longtime limescale monitoring. A solution for the integration of a non-invasive reference sensor system to detect the growing layer thicknesses must be found. The system temperature can be ensured by sufficiently long acclimatization phases in a climatic chamber.

Finally, to integrate this technology into acoustic flow sensor applications, the calibration curves for the flow rate measurement must be extended by limescale layer and temperature sensitivity. These can be derived from further simulations considering different flow velocities, layer thicknesses, and temperatures at the same time. The temperature influence on the propagation times caused by limescale layers will increase if the expected layers become even thicker than currently considered. In this case, the still unknown temperature dependencies of limescale should be investigated and integrated into the simulations.

In summary, this work presents a new method for the online monitoring of temperature and internal layer deposition in Lamb wave-based water meters by means of simulated data. This method will allow for compensating temperature- and layer-induced measurement errors in future flow measurement applications without the need for additional measurement equipment, in turn leading to higher precision in flow measurement, longer product lifetimes, and adapted maintenance intervals.

## 6. Patents

This work refers to patents of the University of Applied Sciences Coburg [41,42] and Diehl Metering GmbH [43].

## Figures and Tables

**Table 1:** Fitting parameters for the calibration curves of the D- and V-wave.

D-Wave		V-Wave	
$a_{1}$	0.002459 µs/µm	$b_{1}$	0.0009874 µs/µm
$a_{2}$	34.47 µs	$b_{2}$	52.37 µs
$c_{1}$	0.005198 µs/K	$e_{1}$	$0.0004417\;µs/K^{2}$
$c_{2}$	34.46 µs	$e_{2}$	−0.04275 µs/K
		$e_{3}$	52.38 µs
